# Effects of Linear Openings in Forests on Temperate Bird Communities

**DOI:** 10.1002/ece3.72466

**Published:** 2025-11-16

**Authors:** Mariela Yapu‐Alcazar, Raphaël Benerradi, Michael Wohlwend, Grzegorz Mikusiński, Ilse Storch, Manisha Bhardwaj

**Affiliations:** ^1^ Chair of Wildlife Ecology and Management University of Freiburg Freiburg Germany; ^2^ LIRMM University of Montpellier, INRIA Montpellier France; ^3^ School for Forest Management Swedish University of Agricultural Sciences Skinnskatteberg Sweden; ^4^ Mammal Research Institute Polish Academy of Sciences Białowieża Poland

**Keywords:** forest roads, functional traits, habitat fragmentation, linear openings

## Abstract

Narrow, unpaved forest roads and paths are a ubiquitous feature of managed forest landscapes worldwide, with the potential to influence bird communities. However, compared to large roads, the effects of structural changes to the understory and canopy generated by unpaved forest roads and paths are less understood. In this study, we investigate the influence of narrow linear openings in the forest caused by forest roads and paths on bird communities in the southern Black Forest, Germany. We surveyed bird communities in four distinct plot types, including two “linear openings”: forest interior, forest paths, forest roads, and forest edges. Forest roads and paths were expected to represent intermediate conditions in terms of openness between interior forests and a forest edge. We aim to understand how these linear openings affect birds' species richness, community composition, and functional traits. Our results show that while species richness remains similar among plot types, the community composition at forest edges differs. The indicator analysis reveals indicator species for each type of plot. In addition, functional traits like body mass and wing shape showed a weak response to the linear openings. The findings suggest that although unpaved forest roads and paths potentially introduce resources and structural modifications in the forest structure, the effect on the birds seems limited compared to pronounced habitat transitions, such as forest edges. These narrow linear infrastructures are often necessary for forest use by humans and can be unnoticeable for birds when carefully planned on a small scale. Nevertheless, forest managers should not overlook broader‐scale effects (e.g., potential habitat loss, predation). Our findings contribute to a better understanding of birds' responses to linear and small‐scale fragmentation introduced by unpaved forest roads and paths. However, more research is needed to distinguish the ecological impacts and management implications for bird communities in temperate managed forests along a gradient of linear openness.

## Graphical abstract

Surveyed bird communities across a gradient of canopy openings (forest interior, paths, roads, and edges) in the Black Forest, Germany. Despite structural differences, bird communities along narrow paths and roads resembled forest interiors more than edges, suggesting these linear features do not function as distinct ecological habitats.

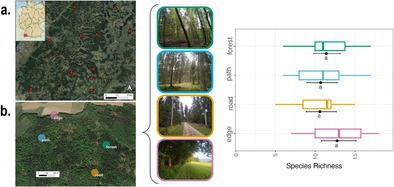

## 1 Introduction

The expansion of road networks has widely impacted forested landscapes and species living in them (Vepakomma et al. 2018). For instance, in temperate and boreal forest environments, road networks of different scales transect these areas, creating artificial corridors across the landscape and linearly breaking the continuity of the forest structure (Riitters and Wickham 2003). The ecological impacts of the disruption of habitat continuity by roads, particularly on wildlife, have been widely studied and can be perceived as either beneficial or detrimental (Morelli et al. 2014). Negative effects of roads on wildlife include: habitat fragmentation (e.g., van der Ree et al. 2007), habitat loss (e.g., Kociolek et al. 2011), increased animal mortality (e.g., Reijnen and Foppen 2006), and pollution, e.g., air, light, noise (e.g., Morelli et al. 2014). However, roads can also increase habitat heterogeneity by providing more foraging opportunities from diversified vegetation (Helldin and Seiler 2003; Morelli et al. 2014; Palomino and Carrascal 2007). Furthermore, in the specific case of birds, roads' associated human structures and marginal vegetation create perching and nesting opportunities (Morelli 2013; Morelli et al. 2014).

Different types of linear infrastructures, including highways, rural roads, and forest roads, each have specific effects on biodiversity (Coffin et al. 2021). While much research has focused on large paved structures with high traffic volumes, unpaved minor forest roads require special attention because of their unique ecological consequences (Coffin et al. 2021; Šálek et al. 2010). On the one hand, the attractiveness of large road research is attributed to the scale of impact on biodiversity (Mammides et al. 2016). Large linear infrastructures, in addition to greatly modifying and fragmenting the landscape, also increase animal mortality and bring a greater flow of humans and pollution (Mammides et al. 2016; Robson and Blouin‐Demers 2013). In contrast, on a smaller scale, unpaved forest roads linearly fragment contiguous habitats, create openings in the canopy, alter ground vegetation, and form edges that can modify the forest structure (Arjmand et al. 2023). In managed forests, two kinds of unpaved roads can be identified based on their size: larger forest roads and smaller roads like skid trails and foot paths. The former provide access for heavier vehicles, while skid trails and paths are used for the transport of logs or by hikers (LWF 2017; Mercier et al. 2019). Such infrastructures cause linear openings, altering the ground due to compaction or trampling and altering light availability and microclimate, with stronger effects on bigger roads but still found in smaller roads. The disturbance results in a modified vegetation structure and composition, creating intermediate habitat characteristics distinct from forest interior and forest edge conditions (Marchais et al. 2024; Zhou et al. 2020). These characteristics differ from those of more pronounced disturbances, such as intensive clear‐cutting with large canopy openings, but still play a significant role in forest ecosystems (Boston 2016). The openness gradient covering forest interior–path–roads–edge may reflect an interface of structural complexity, such as variation in tree density and ground vegetation, which can shape resource availability and influence biodiversity, including bird communities (Šálek et al. 2010). Birds, particularly forest specialists, could be negatively affected due to their dependence on undisturbed interior environments for nesting or foraging (Huhta et al. 1999). Nevertheless, generalist species might benefit from new foraging possibilities associated with openings in the ground and in the canopy resulting from forest roads (da Silva et al. 2017; Kroeger et al. 2022).

In some Central European forests, unpaved forest roads with low traffic volume can be considered environments of higher structural complexity in comparison to the surrounding forest stands. In areas of intensively managed forests, it has been found that these linear infrastructures could lead to an increase in the abundance and diversity of trees and shrubs compared to the forest interior (Klimo and Kulhavy 2006). When embedded within a contiguous forest habitat, these narrow openings create corridors within the forest matrix, and although they are not the true forest edge by definition, they can still produce edge‐like effects by modifying the vegetation structure and microclimate along the road (Šálek et al. 2010). Birds, for instance, may utilize the edges of forest roads for foraging (Ries et al. 2004; Yahner 1988), highlighting the significance of these areas as environments with specific resources. The landscape supplementation hypothesis suggests that such modified areas, like the ones introduced by the roads, can support biodiversity by providing different foraging opportunities within the larger forest matrix (Dunning et al. 1992; Šálek et al. 2010). However, increasing structural openness in the forest (e.g., reductions in vegetation cover or tree density) has risks as well, like a rise in bird brood parasitism and nest predation (Fletcher and Hutto 2008; Johnson and Temple 1990). In fact, it has been demonstrated that the closer to the forest edge, the higher the predation risks, which indicates a greater abundance of nest predators or brood parasites (Chasko and Edward 1982; Cox et al. 2012).

The creation of linear openings impacts bird diversity and influences interspecific competition via traits related to size and mobility (Newbold et al. 2013; Robertson et al. 2013). More open habitat types often represent areas with altered resource availability, where species with greater body mass, usually associated with competitive dominance, may be better equipped to exploit various resources. Conversely, smaller species may struggle in these environments because of strong competition from larger species (Brown 2007; Pfeifer et al. 2017). The traits related to mobility would considerably affect how species navigate through fragmented habitats, affecting their capacity to reach and utilize available environments in line with the habitat complexity–maneuverability constraints hypothesis (Polo and Carrascal 1999). Birds with a higher Hand‐wing Index (HWI) and body mass, which imply greater flight capacity and efficiency, would be more adapted to move along the linear openings, between habitat margins and adjacent forest patches, allowing them to use resources from several environments. On the other hand, less mobile species (with lower HWI) and small size would have reduced resource availability and dispersal options, thereby restricting their ability to benefit from alternative, more open habitats (Claramunt et al. 2012; Sheard et al. 2020).

**FIGURE 1:**
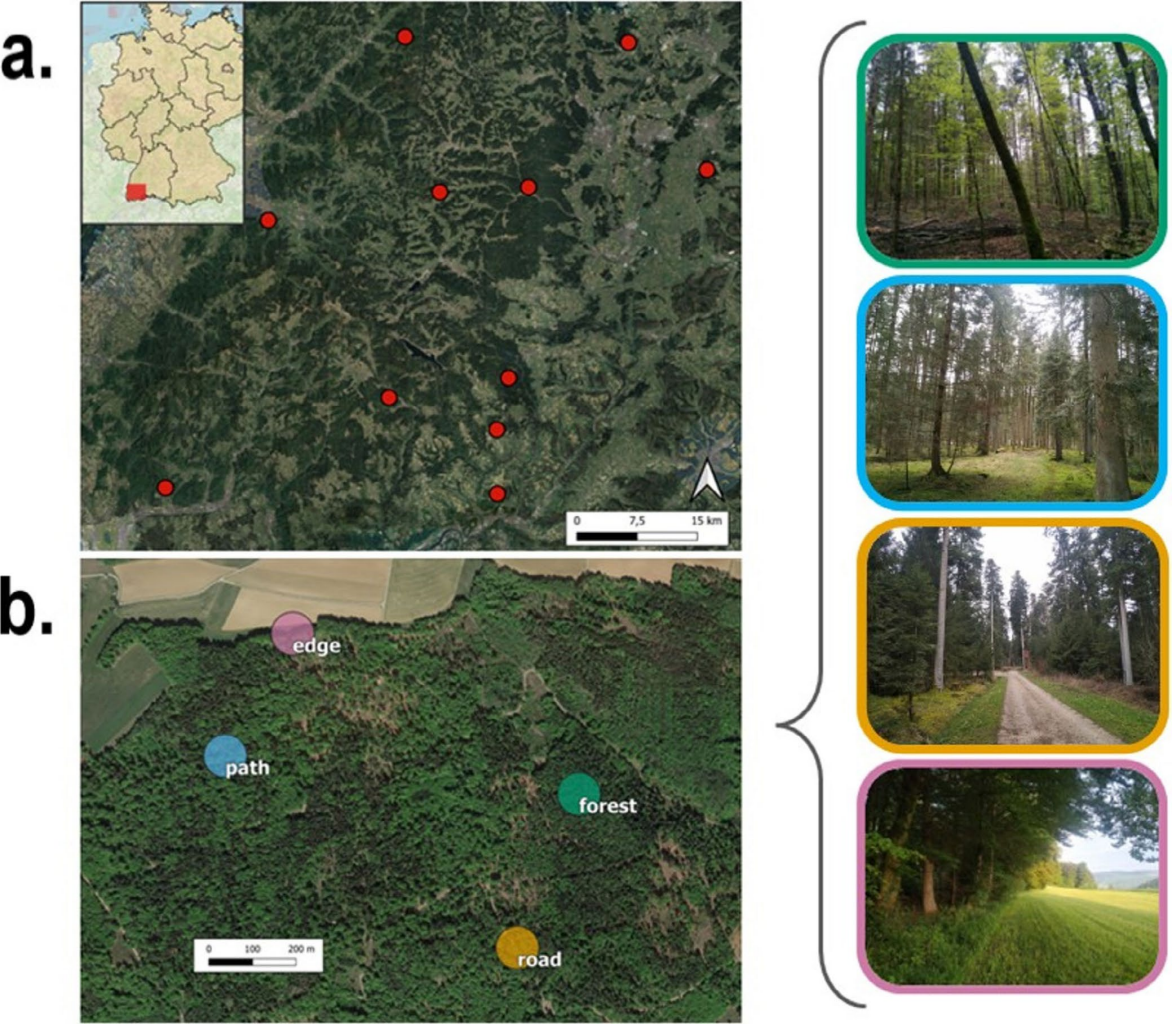
Study area and sample design in the Southern Black Forest showing 11 sites, each containing four plots. a. distribution of the 11 study sites in the Southern Black Forest, SW Germany. b Within each site, four plots represent the four types of sampling units: Forest plot (top right), path plot (second from top right), road plot (second from bottom right), and edge plot (bottom right).

In managed temperate forests, the research concerning the impact of unpaved forest roads on birds is very limited (Helldin and Seiler 2003; Palomino and Carrascal 2007; Wu et al. 2023). Šálek et al. (2010) reported that low‐traffic forest roads in Czech secondary production forests managed mostly by rotation forestry can increase bird species richness and diversity by creating edge habitats. To see if such influence applies to unevenly aged continuous‐cover production forests, we focus on the southern Black Forest, Germany, where forest management practices aim to preserve biodiversity and promote natural regeneration along with production goals (Bauhus et al. 2013; Storch et al. 2020). Our study assesses two key response dimensions from birds to the linear openings in the forest created by linear infrastructure: community response and trait response. For the community response, we evaluate whether species richness, diversity, and community composition of bird communities differ among sites with different levels of linear openings—namely, forest interiors, paths, forest roads, and edges (see Figure 1). We predict that forest roads, followed by paths would allow for a more diverse and differently composed community than forest interiors (P1). In addition, we evaluate functional traits connected to mobility, body mass, and HWI, and how these influence the response of birds to linear openings. We predict these traits will exhibit more extreme values in species with a favorable response to linear openings (P2). Therefore, our study assesses whether linear openings created by forest roads and paths serve as complementary habitats for forest birds. Our questions align with the landscape supplementation hypothesis (Dunning et al. 1992) and the habitat complexity–maneuverability constraints hypothesis (Polo and Carrascal 1999) to reveal the role of these environments in shaping bird community dynamics in temperate forest ecosystems. By answering these questions, we aim to provide insight into the bird communities' responses to these fine‐scale disturbances. We also expect that our findings can provide useful information in the framework of biodiversity conservation in managed forests, particularly in regions where roads and paths are a permanent feature of the forest landscape.

## 2 Methods

### 2.1 Study Area and Site Selection

We conducted this study in the southern Black Forest region in Germany (47.66°–48.17° N, 7.72°–8.64° E) within temperate mixed montane forests located in a low mountain region in Central Europe (altitude 516–1120 m). For biodiversity conservation, these production forests are managed under close‐to‐nature forestry guidelines, resulting in a mixed, uneven‐aged, and structurally diverse forest with continuous canopy cover (Bauhus et al. 2013; Kändler and Cullmann 2015). Since we were interested in exploring how different linear openings affect bird communities, we surveyed birds on four plot types: forest interior, forest paths, forest roads, and forest edges (Figure 1, Table 1). The linear openings were initially detected using geospatial datasets in QGIS based on the types of roads from Geofabrik (Geofabrik 2023) (Appendix 1). Based on this spatial information, candidate plots were pre‐selected and subsequently validated through initial site visits to confirm their characteristics visually in the field. Plots were spatially clustered into 11 distinct sites (based on the broader research framework established by the Research Training Group “Conservation of Forest Biodiversity in Multiple‐use Landscapes of Central Europe”; Figure 1; Storch et al. 2020). Sites were at least 5 km from each other, and plots within sites were at least 300 m apart to ensure independent sampling of bird communities (Bibby et al. 2000; Sutherland 2006), except at one site, where geographical constraints meant the plots were 200 m apart instead. The sites were far from other openings caused by natural (e.g., windthrow and bark beetle) or anthropogenic factors (e.g., logged areas).

**TABLE 1:** Description of plot types within each site. We surveyed 11 of each plot type.

Plot type	Description
Forest plots	Plots in the forest interior, with a canopy cover > 80% within a 50 m radius, at least 200 m away from paved roads and forest edges, and at least 100 m away from any unpaved road or path. From now on, referred to as “forest”.
Path plots	Plots centered on linear clearings in the forest that are less than three meters wide and do not fully open the canopy (e.g., wide pedestrian trails or skidding roads), at least 200 m away from paved roads and forest edges, and at least 100 m away from roads. The ground along the path should have vegetation, but not as dense as in the forest interior. From now on, referred to as “path.”
Road plots	Plots centered on gravel forest roads over five meters wide and create a canopy opening. The ground along the road has no vegetation on the ground and is at least 200 m away from paved roads and forest edges. From now on, referred to as “road.”
Edge plots	Plots centered on forest edges, at the interface between forest and meadow, pasture, or cropland, 200 m or more from road. From now on, referred to as “edge.”

### 2.2 Bird Survey and Functional Traits

We used fixed‐radius point counts to assess the composition of bird communities at each plot. From the center of the plot, an observer recorded a count of each species seen or heard in a 50 m radius over four consecutive five‐minute intervals (Balestrieri et al. 2017; Bibby et al. 2000; Sutherland 2006). After each survey, we assigned the abundance of each species on the plot according to the maximum abundance recorded in any of the 4 five‐minute point count intervals. Each plot was visited twice, with at least seven days between the two visits. For the first visit round, we started with the five sites at the lowest elevations to avoid traces of snow. With the remaining sites, we randomly chose the order of the visits to the sites and the order of the plots within the site (*random. shuffle* module of the site IDs and the plots) in Python (van Rossum and Drake 2009). We always surveyed all four plots within a site on the same day. We conducted bird surveys between sunrise and midday and during the breeding season (April and May) in 2023 (according to Balestrieri et al. 2017). In total, we surveyed 44 plots (4 plots × 11 sites), each visited twice, resulting in 88 point count surveys and 1388 individual bird records belonging to 47 species (Appendix 2).

To assess the impact on the functional traits, for each species observed during the surveys, we extracted the average HWI and body mass from the AVONET database (Tobias et al. 2022). For each plot visit, we defined the average body mass and the average HWI observed as the mean of the HWI of the species, weighted by the relative abundance of each species observed during the visit.

### 2.3 Statistical Analysis

First, we utilized species richness and the Shannon–Wiener Index (Pielou 1966; Shannon 1948) as indicators to examine the variations in bird communities across different plot types. Species richness provides an easy count of species present, reflecting an area's overall biodiversity. Conversely, the Shannon–Wiener Index considers both species richness and evenness, providing a deeper understanding of how individuals are distributed among the species found (Magurran 2003; Whittaker 1972). To compare these indicators across various plot types, we employed generalized linear mixed models (GLMMs), using a Poisson distribution for species richness and a Gamma distribution for the Shannon–Wiener Index, including a random effect for site ID. We conducted Tukey's HSD post hoc tests to assess the mean differences of each metric across the plot types based on these statistical models (Zar 2010). To further investigate the differences in species composition among the plot categories, we applied the Jaccard Index of Dissimilarity calculated from abundances, defined as 2B/(1 + B) (Oksanen et al. 2013), where B represents Bray–Curtis dissimilarity (Jaccard 1912). We explored dissimilarities in community compositions at each plot using Non‐Metric Multi‐Dimensional Scaling (NMDS) analyses (Clarke 1993), based on the relative distance between plots according to the Jaccard Index. We then conducted an Analysis of Similarity (ANOSIM) based on the NMDS representation to test for differences between the four plot types, considering the differences between bird communities per survey (Clarke 1993). An Indicator Species Analysis was also conducted to find species that could explain the differences between groups. This method estimates the strength of species associations with some types of plots (de Cáceres and Legendre 2009). Finally, we compared the body mass and HWI, both abundance‐weighted, between the types of plots by using GLMMs with Gamma distributions and applying Tukey's HSD post hoc tests on these models, similar to those for the Shannon‐Wiener Index.

We conducted all analyses in R version 4.2.2 (R Core Team 2023), using the packages vegan (version 2.6.10) for biodiversity indices and ANOSIM (Oksanen et al. 2013), multicomp (version 1.4.28) for Tukey's tests (Hothorn et al. 2016), indicspecies (version 1.8.0) for Indicator Species Analysis (De Caceres et al. 2016), and lme4 (version 1.1.37) for GLMMs (Bates et al. 2014).

## 3 Results

**FIGURE 2:**
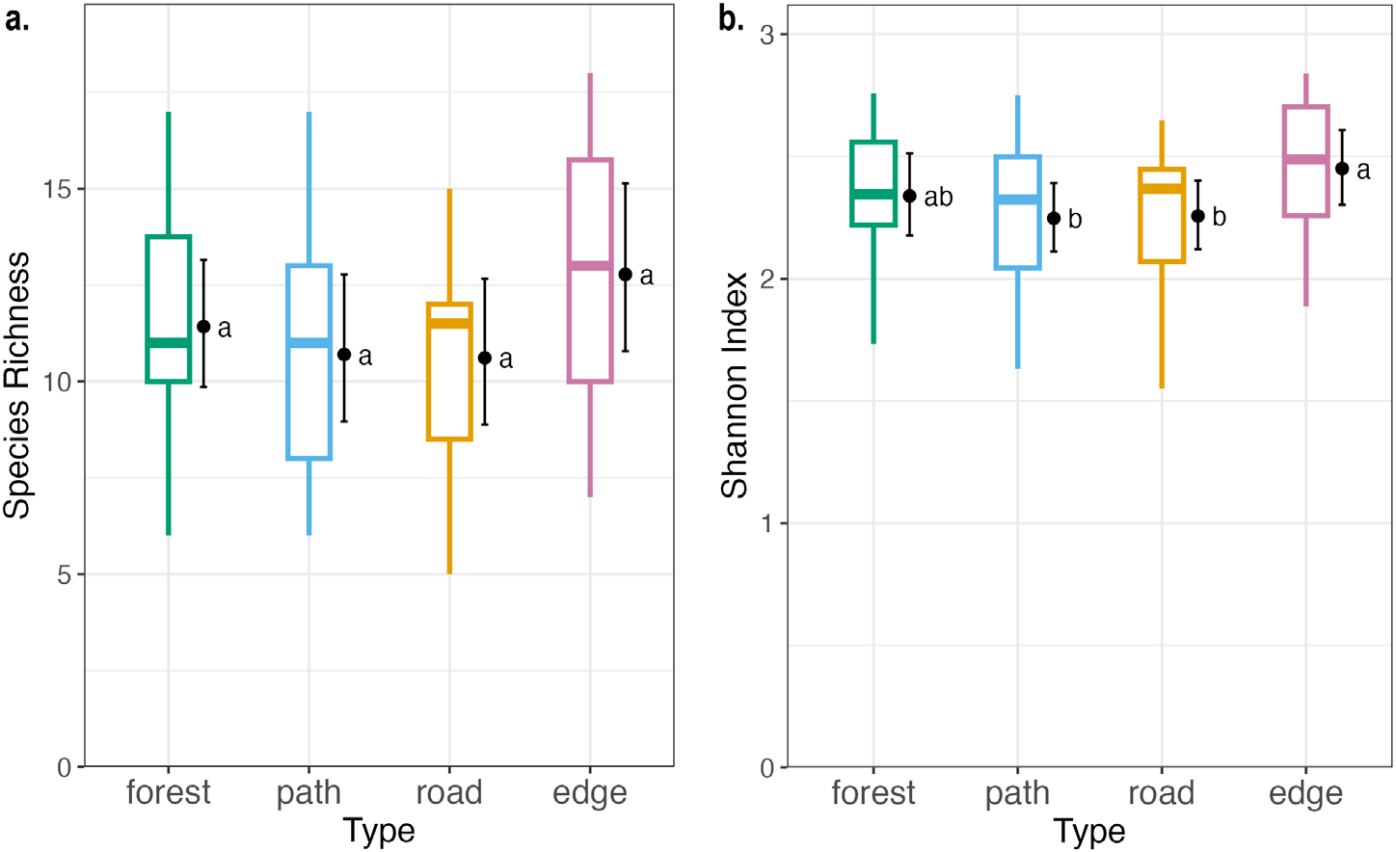
GLMs followed by Tukey's post hoc tests for (a) Species richness and (0). Shannon–Wiener Index across different plot types. Statistical analysis included generalized linear mixed models (species richness ~ plot type using Poisson regression; Shannon–Wiener Index ~ plot type using Gamma regression) with site ID as a random effect. Boxplots display the observed indices, and black dots and error bars represent estimated marginal means with 95% confidence intervals for each plot type. Letters “a” and “b” next to the bars indicate significant differences between groups, where groups sharing the same letter are not significantly different from each other. Note that the estimated marginal means (dots) can be different from the medians (bar in the boxplots) in the observed indices.

Average species richness tended to be highest in edge plots and higher in forest plots than in path and road plots; however, these differences were not statistically significant (*p* > 0.05 for all combinations; Figure 2a). In contrast, the Shannon–Wiener Index was significantly lower in path and road plots compared to edge plots (*p*_edge‐path_ = 0.033, *p*_edge‐road_ = 0.048), but not significantly different between paths and roads (Figure 2b). Also, the index at forest plots tended to be higher than at paths and roads but lower than at edge plots, though these differences were not statistically significant (Figures S1 and S2).

**FIGURE 3:**
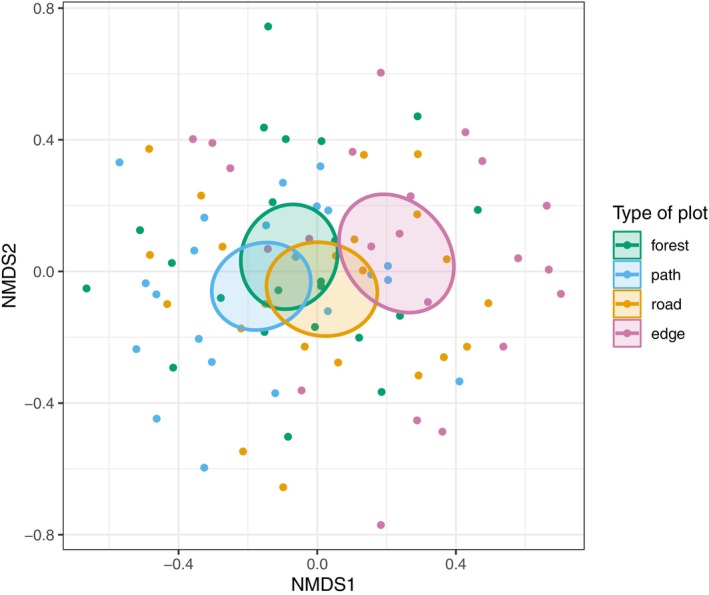
NMDS representation of the visits colored by type of plot, considering the Jaccard Index of Dissimilarity. The ellipses represent the 95% confidence intervals around the centroids for each plot type, indicating the regions around which most bird communities of each plot type are expected to occur. Appendix 3 incorporates species in this NMDS representation.

Overall, the community compositions in forest, path, and road plots were relatively similar to one another (Figure 3). Community composition in edge plots differed the most from the other plot types (Table 2, Figure S3, Figure S4). The lack of clear separation between the 95% confidence ellipses for the centroids suggests that while species compositions slightly differ between plot types, the overall dissimilarity is not strong enough to form visually distinct clusters. Still, we can distinguish species that tend to cluster around specific plot types (Table A1 in Appendix 2, Appendix 3). These compositional shifts support the more detailed species‐level patterns described in the Indicator Species Analysis.

**TABLE 2:** Results of ANOSIM tests for each combination of plot types are compared.

Types of plots compared	Statistic *R*	*p*
Forest—Path—Road—Edge	0.082	0.001***
Forest—Edge	0.169	0.001***
Path—Edge	0.167	0.002**
Road—Edge	0.078	0.014*
Forest—Path—Road	0.027	0.113
Forest—Path	0.038	0.118
Path—Road	0.024	0.199
Forest—Road	0.020	0.234

*Note:* Plots that are significantly different at *α* = 0.05 are indicated with *, at *α* ≤ 0.01 with **, and at *α* ≤ 0.001 with ***. Diagnostic ANOSIM plot available in Figure S5.

**FIGURE 4:**
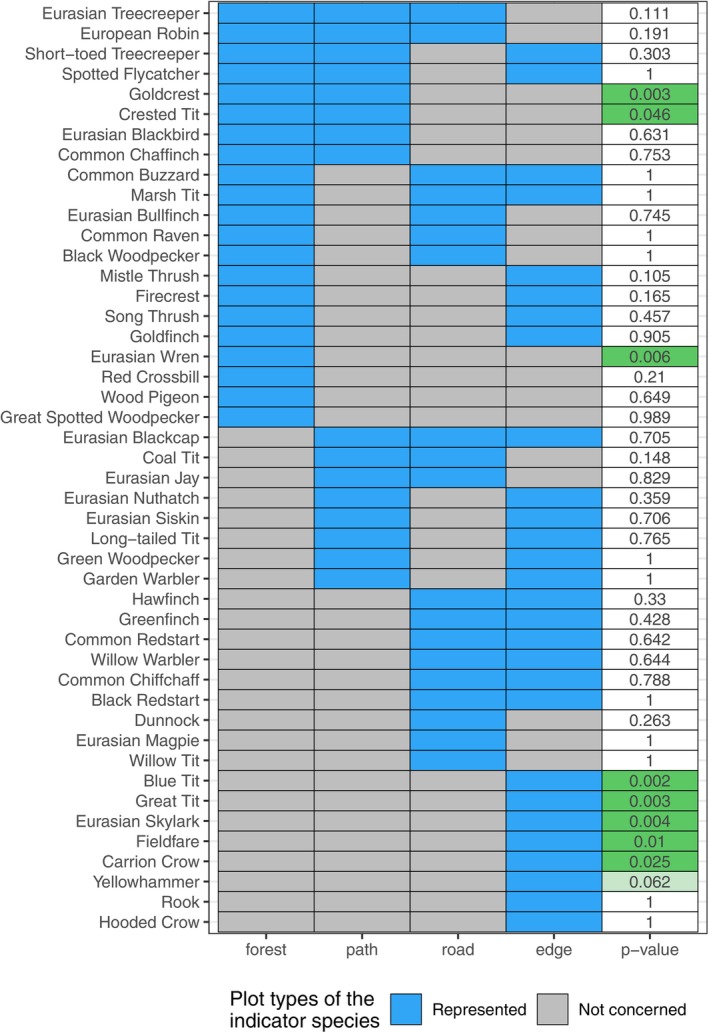
Summary of the Indicator Species Analysis. Each species is associated with a set of types of subplots, and a *p*‐value testing the significance of the association. Green boxes represent species considered indicators of the represented plots (blue boxes).

With a significance threshold of 0.05, the Indicator Species Analysis reveals eight Indicator Species. For the forest plots, the Eurasian Wren (*p*‐value = 0.006) represents the only indicator species. For forest and path, Goldcrest (*p*‐value = 0.003) and Crested Tit (*p*‐value = 0.046) are the two indicator species. The indicator species for edges are five: Blue Tit, Eurasian Skylark, Great Tit, Fieldfare, and Carrion Crow (*p*‐values: 0.002, 0.003, 0.004, 0.01, 0.025, respectively) (Figure 4). Eurasian Skylark, Fieldfare, Carrion Crow, Yellowhammer, and Rook were observed only on edges. Except for four species observed exclusively in non‐edge plots (Black Woodpecker, Eurasian Magpie, Common Raven, Willow Tit; only 1–2 records per species), all others were also present on edges.

### 3.1 Functional Traits Response

**FIGURE 5:**
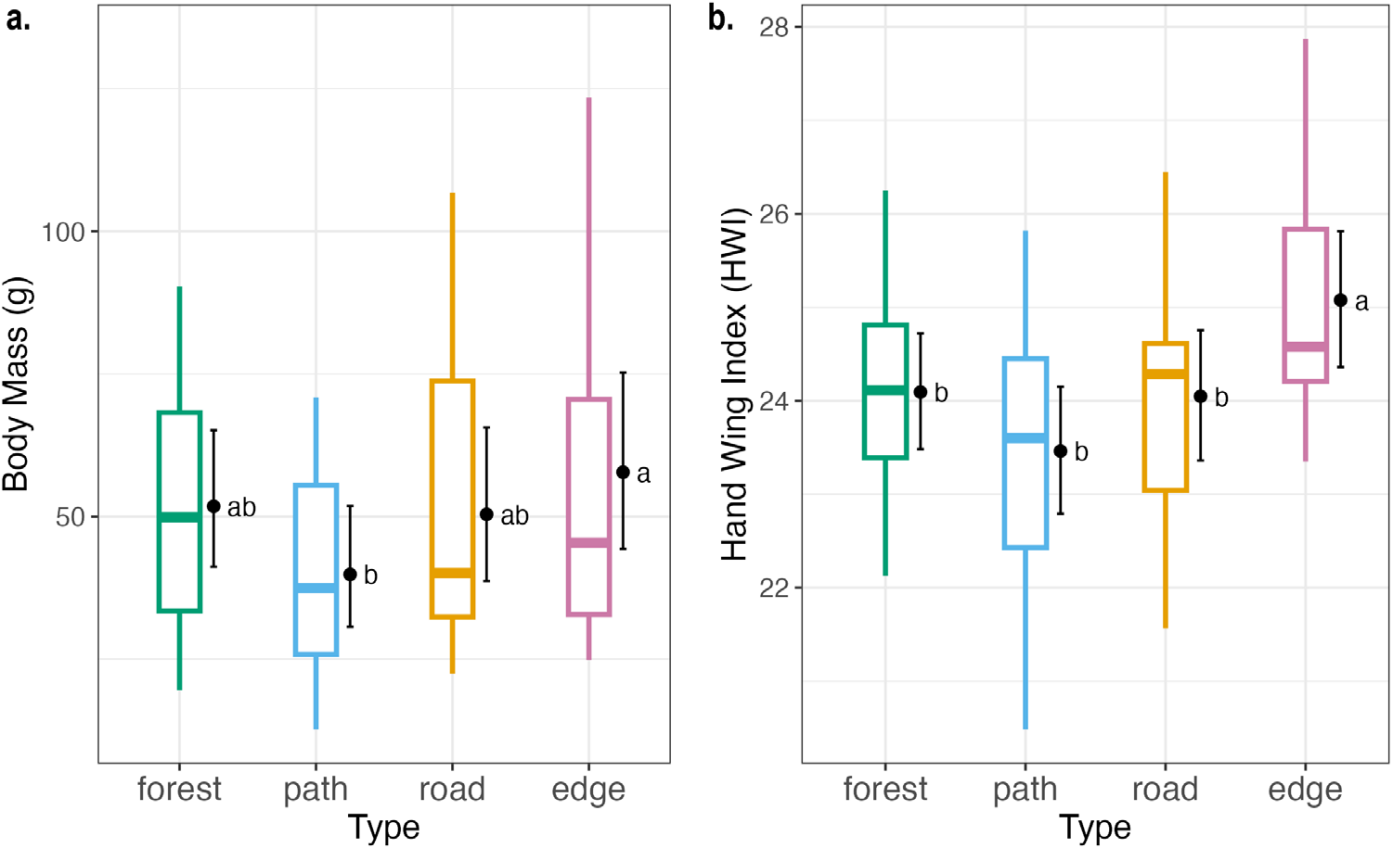
Gamma regressions followed by Tukey's test on the weighted mean of the (a) body mass and (b) Hand‐wing Index (HWI) on a visit, depending on the type of plot, with the site ID as a random effect. Boxplots are the observed indices. Dots and error bars represent estimated marginal means with a 95% confidence interval for each type of plot. Letters “a” and “b” next to the bars indicate significant differences between groups, where groups sharing the same letter are not significantly different from each other. Note that the estimated marginal808 means (dots) can be different from the medians (bar in the boxplots) in the observed indices.

The weighted mean body mass tended to be lower in path plots compared to the other plot types (Figure 5), but the relationship was only significant when compared to edge plots (*p*_edge‐path_ = 0.03). The average HWI was significantly higher in edges than in other types of plots (*p*_edge‐path_ < 0.001); however, it did not differ among forests, paths, and roads (*p*_forest‐path_ = 0.27, *p*_forest‐road_ = 0.99, *p*_path‐road_ = 0.34) (Appendix 2, Figure S5).

## 4 Discussion

In this study, we investigated the understudied ecological effects of linear openings in the forest created by forest roads and paths on bird communities. Our results show that species richness was similar across forest interiors, paths, roads, and edges. However, we found differences in community diversity (Shannon–Wiener Index) and specific traits like body size and Hand‐wing Index (HWI). Overall, bird species and trait diversity on paths and roads were more similar to forest interiors than forest edges. These results suggest that in spite of the structural differences due to the linear openings, forest roads and paths do not act as distinct habitats like the edge itself. Rather, birds respond to these areas as embedded within the forest. Our findings reveal to what extent linear openings influence bird communities, offering valuable insight into how narrow forest roads affect biodiversity in temperate managed forests.

### 4.1 Limited Impact of Linear Canopy Openings

We predicted (P1) that bird communities would be affected by linear openings, with an increase in richness and diversity in these sites similar to the edges. While our results show that bird communities are slightly less diverse along forest roads and paths, they do not significantly differ from the forest interior. The homogenous species richness across the four plot types supports the landscape supplementation hypothesis (Dunning et al. 1992), and suggests that birds in the Black Forest utilize the resources of the different plot types rather than being restricted to a specific one. Furthermore, the Shannon–Wiener Index and ANOSIM analysis revealed that only forest edge communities differed significantly from the other plot types. Thus, in our study, the expected impact of linear openings was restricted to the edges and was absent in plots with narrower openings.

The pattern of limited sensitivity to structural differences and human‐induced disturbance in forests has been previously reported in the context of European forest bird communities (Basile et al. 2021; Lelli et al. 2019; Matuoka et al. 2020). This resilience to environmental changes is a notable characteristic among generalist species, which are particularly dominant in temperate managed European forests such as the Black Forest. Several bird species in our study area have forests as a main habitat but also feed in nearby open areas (e.g., thrushes) (Chiatante 2022; Najmanová and Adamík 2009), demonstrating flexible habitat use. European temperate forest birds appear more adaptable to environmental changes than other bird guilds worldwide, likely reflecting a legacy effect of centuries of habitat alteration by human activity (Salisbury et al. 2012). Biodiversity in the Black Forest region has been shaped by generations of land‐use practices, including intense forestry (Brandl 2006), and bird species composition is expected to be fairly homogeneous in this production‐oriented forest system (Basile et al. 2021; Freemark and Kirk 2001). Moreover, like in all central European forests, birds in our plots are mostly residents or short‐distance migrants, with a few tropical migrants (Appendix 1) (van Turnhout et al. 2010; Villard and Foppen 2018). Hence the weak response to linear opening in our study may be due to the fact that our study area is dominated by species that are highly resistant to human activity (Reif et al. 2022), whereas it has been reported that the effects of habitat perturbation tend to be more pronounced in long‐distance migrants (Gregory et al. 2007). Some reduction in the Shannon–Wiener Index values that we observed in paths and forest roads, compared to forest interiors, may be because narrow corridors frequently can act as ecological traps for birds, increasing predation and brood parasitism (Gates and Gysel 1978; Henle et al. 2004; Rich et al. 1994). Summarizing we suggest that the legacy of management and human alteration in the Black Forest may have curated a homogenous community composition, dominated by generalists with flexible habitat use, which likely buffers against structural changes such as linear openings in our study. Therefore, the real impact of forest structural changes on birds must be assessed together with the regional and historical context.

### 4.2 Scale of the Linear Canopy Openings and Bird Mobility

The lack of response to canopy openings is also reflected in our functional trait results. We did not observe the gradual increase in body mass along the gradient of canopy openness (from forest interior through paths and forest roads to edges), that we expected. We expected body mass to be higher on forest roads and paths compared to interiors, where the canopy openness allows more mobility than in forest interiors. Contrastingly, we found that the interior of the forest has larger birds. Similarly, HWI does not show significant changes in the openness gradient, contradicting the habitat complexity–maneuverability constraints hypothesis (Polo and Carrascal 1999). The increase in HWI is only found at the edges, where birds should certainly be adapted to longer flights (Claramunt et al. 2012; Sheard et al. 2020). These findings suggest that only large openings affect these traits, and therefore, forest roads and paths in our study area do not reach the threshold width of openness necessary to influence bird mobility traits.

The effects of the level of openness width have been documented in previous studies as a considerable factor (e.g., Šálek et al. 2010; Wu et al. 2023). In both studies, where the openings caused by forest roads are also evaluated, the stronger effect on bird communities is reported the wider the road is. While openings like unpaved roads can increase the number of potential niches (Kociolek et al. 2011; Sander and Tietze 2022), similar types of openings on a larger scale bring fragmentation and habitat loss, reducing the ecological value for birds (Rich et al. 1994). In our case, the openings were narrow, visually estimated at 3–5 m width, contrasting with the opening scale at the forest edge. This contrast emphasizes the importance of opening size in shaping bird communities, highlighting that narrow openings within a forest matrix may function differently from non‐linear openings. In the primeval Białowieża Forest, even relatively small gaps in the canopy caused by tree fall had significantly different bird communities in comparison to closed forest (Fuller 2000). However, despite the heterogeneity created by the introduction of openings, highly mobile species like birds might still perceive a continuous habitat where they cross the small breaks (Lord and Norton 1990). This reinforces the findings of the traits and leads us to conclude that the linear canopy openings in our study area would function primarily as movement corridors within the forest rather than novel environments with unique structural features like those on the edge for birds. However, while the overall community composition and mobility traits remained relatively stable across narrow openings, certain species exhibited distinct responses along the gradient. It is worth pointing out that our findings contrast with what occurs in low‐latitude forests. In tropical forests, the contrast between the forest, unpaved roads, and adjacent vegetation is sharper, causing a more pronounced edge effect and more noticeable changes in bird communities (Laurance 2004).

The Indicator Species Analysis identified bird species associated with three plot types, showing that changes in the level of structural openness can influence habitat preferences of specific species. Furthermore, the species identified by this analysis follows our expectations for the functional traits, particularly for the HWI. The Eurasian Wren emerged as an indicator of forest interior and has the lowest HWI. This species' foraging and nesting heavily depend on forests with abundant understory (Piechnik et al. 2020; Wesołowski 2007). At the same time, in forest environments, it is a species sensitive to human presence (Kroodsma et al. 2020), which might explain their reduced presence in paths and roads where human activity is more frequent (e.g., hikers or forest operations). On paths, the Goldcrest is more common, consistent with its known habitat use, and its HWI is higher than that of the Wren. This species forages by flying between branches and moving vertically up and down the trees (Martens and Päckert 2020), a behavior that is facilitated in areas with still sufficient vertical structure, like forest interior and paths, but reduced in roads and edges. The edge area is the one that shows the highest number of indicator species. All of them, regarding habitat requirements prefer open forest or farmlands, and seldom use or avoid completely dense forest. The latter includes the Fieldfare (Collar 2020) and the Skylark (Campbell et al. 2011). Both species also have the highest HWI among the indicator species, which is suitable for flying in open areas. Also, the Yellowhammer, which is a species linked to farmland or early‐succession openings in the forest (Bakx et al. 2020), was recorded only on the forest edge indicating that studied forest roads did not provide any suitable habitat for the species.

### 4.3 Limitations and New Directions

Our findings attempted to clarify the ecological impacts of linear openings in the canopy created by low‐traffic roads and offer a glimpse of the bird communities' responses along an openness gradient in temperate managed forests. However, we noted that three methodological limitations should be considered when interpreting or extrapolating these results. First, the results we provide are restricted to the breeding season during one year. On the one hand, even though most of the bird research is done during the breeding season, bird dynamics in temperate forests vary with seasonality throughout the year. During early spring or winter, the linear openings in forest roads may play different ecological roles as they frequently act as early warm sunny spots during snowmelt, exposing resources important for birds replenishing gastroliths (Keyser et al. 2023; Resano‐Mayor et al. 2019). Also, small fruiting trees like Rowan are more common along roads and are potential food resources before winter (Askeyev et al. 2024). Second, despite consistently selecting plots based on spatial classification and subsequent field validation, there are site‐specific factors such as slope and orientation that we did not directly evaluate, and that could influence microclimatic conditions, vegetation structure, and resource availability, potentially shaping bird community responses along linear openings in the canopy (Ogée et al. 2024; Wang et al. 2014). Finally, as previously mentioned, a factor to consider that could strengthen our findings is incorporating wider linear openings as Šálek et al. (2010) and Wu et al. (2023). Observations over a broader gradient in terms of width may give better inferences of the impacts of linear openings on bird fauna. To address these challenges, we encourage future research regarding linear openings due to forest roads to address the aforementioned variables, particularly comparing different widths of linear openings. Where possible, before‐and‐after control‐impact (BACI) experimental designs may provide a powerful experimental way of measuring the environmental impact of these infrastructures in the forest (Klein et al. 2022; Roedenbeck et al. 2007). Such design could be applied to both planned new forest roads and forest roads that are removed as a forest restoration measure (Switalski and Nelson 2011; van der Ree et al. 2015).

### 4.4 Management Implications

Any infrastructure that disrupts the continuity of the forest and its biodiversity must always be considered when managing forest biodiversity (Kroeger et al. 2022). In our particular case, an area with a long history of management, it is vital to understand the dynamics of these linear structures in order to incorporate changes and improvements into management plans. Although not conclusive, our results provide a glimpse of what is happening with these linear infrastructures in the Black Forest and their impact on birds. The limited effect could support the integration of low‐impact structures, such as unpaved trails, to continue silviculture activities or recreation activities. However, these infrastructures must be installed and maintained, considering that the forest matrix should remain as minimally fragmented as possible. This means avoiding abrupt changes such as those that occur at the edge of the forest, where farmland and agricultural land would mean a shift in bird composition. In addition, we consider it important to pay attention to prevent these small roads from expanding due to a lack of control in the long term, as can happen (Laurance et al. 2014). For instance, actively work against an increase in forestry activities or even an increase in the use of mountain bikes (Bayne et al. 2022).

Finally, it should be noted that restoration and rewilding projects aim to remove minor roads as a solution to habitat fragmentation (Ascensão et al. 2019; Clevenger 2005). However, eliminating minor forestry roads in temperate forests is, according to our study, unlikely to create more forest interior habitats and directly boost bird diversity. Several factors should also be considered, such as the speed of the forest regeneration (St‐Pierre et al. 2021), specific responses of birds, and landscape connectivity (Ring et al. 2024).

## 5 Conclusion

Our findings indicate that although forest roads and paths may induce structural heterogeneity, their role is established more as supplementary areas to the main bird habitat within or at the edge of the forest, and not a distinct one. The weak response of the bird communities to these linear structures indicates that birds show a clear response rather to a more abrupt habitat transition, such as forest edges. Although some species respond to linear openings, we found no consistent evidence that these features enhance bird diversity or broadly mimic edge effects at the community level. The absence of significant trait‐based differences further suggests that narrow forest roads and paths do not impose distinct selective pressures on the community, but it does on some species. The scope of our work allows us to clarify a small part of the bird responses involving unpaved forest roads and provide useful information regarding forest roads to forest managers on how to perceive these linear infrastructures. Future research could examine the interplay between habitat connectivity, vegetation structure, and road and canopy width openings to further disentangle their effects on bird communities in similarly managed landscapes.

## Author Contributions

**Mariela Yapu‐Alcazar:** conceptualization (equal), investigation (equal), methodology (equal), supervision (equal), writing – original draft (equal). **Raphaël Benerradi:** conceptualization (equal), data curation (lead), formal analysis (lead), investigation (equal), methodology (equal), visualization (lead), writing – original draft (equal). **Michael Wohlwend:** conceptualization (equal), methodology (equal), supervision (equal), writing – original draft (equal). **Ilse Storch:** funding acquisition (lead), writing – original draft (supporting). **Grzegorz Mikusiński:** writing – original draft (equal). **Manisha Bhardwaj:** conceptualization (equal), methodology (equal), supervision (lead), writing – original draft (equal).

## Conflicts of Interest

The authors declare no conflicts of interest.

## Supporting information


**Figure S1:** Bird abundances per plot types analysis. a. Tukey's HSD post hoc tests on the total abundance per type of plot, with the plot as a random effect. The model uses a Poisson regression. Boxplots are the observed as total abundances. Error bars represent (estimated marginal) means with 95% confidence interval for each type of plot the Tukey's HSD post hoc test shows no effect of the type of plots on the total abundance. Letters “a” and “b” next to the bars indicate significant differences between groups, where groups sharing the same letter are not significantly different from each other. Note that the estimated marginal means (dots) can be different from the medians (bar in the boxplots) in the observed indices. b. Pairwise comparisons in the Tukey's HSD post hoc test on the total abundance depending on the type of plots.
**Figure S2:** Pairwise comparisons in Tukey's HSD post hoc depending on the plot type on the a. Species richness and b. Shannon Index (associated with Figure 2a,b, respectively).
**Figure S3:** Tukey's HSD post hoc test on the effects of the pair of types of plots on the dissimilarity with the Jaccard Index of Dissimilarity on presence/absence data, with the pair of plots as a random effect, using normal distributions (ANOVA with mixed effects). Boxplots are the observed Jaccard dissimilarities. Red dots and error bars represent (estimated marginal) means with 95% confidence interval per group. Letters “a” and “b” next to the bars indicate significant differences between groups, where groups sharing the same letter are not significantly different from each other.
**Figure S4:** Ranks in the ANOSIM between points among groups compared to the overall ranks plot.
**Figure S5:** Pairwise comparisons in the Tukey's HSD post hoc test on the weighted mean on the a. Body Mass and b. HWI for each pair of plot types.

## Data Availability

The data that support the findings of this study are openly available in the Figshare repository at https://figshare.com/s/2a4de30d3f02750df921.

## Appendix 1

Study plots selection. The linear openings were initially detected using geospatial datasets from road type classification Available in Geofabrik. Forest interior (Green), light blue (Paths), Forest roads (Yellow), and edge (Pink).

## Appendix 2

**TABLE A1:** List of abundance and number of presences per species per type of plots.

Species (common name)	Abundance					Presence					Bird traits and features		
	Forest	Path	Road	Edge	Total	Forest	Path	Road	Edge	Total	Mass (g)^a^	Hand wing index (HWI)^a^	Migratory status^a^
Common Chaffinch	42	40	37	36	155	21	21	21	21	84	23.81	27.6	Short migrant
Coal Tit	29	36	35	26	126	18	22	21	17	78	9.2	21.9	Short migrant
European Robin	32	31	32	24	119	19	22	20	17	78	17.7	19.9	Short migrant
Eurasian Blackbird	33	30	28	26	117	20	19	21	18	78	102.73	23.2	Short migrant
Goldcrest	29	35	18	16	98	15	19	12	14	60	5.54	22	Short migrant
Eurasian Wren	30	20	19	15	84	21	16	14	14	65	9.74	16.2	Short migrant
Eurasian Blackcap	16	20	24	21	81	11	13	15	15	54	16.7	27	Short migrant
Common Chiffchaff	19	17	21	22	79	14	14	15	17	60	8.3	18.4	Tropical migrant
Mistle Thrush	16	10	11	19	56	13	9	10	16	48	117.37	35.1	Short migrant
Great Tit	10	9	11	24	54	7	9	9	19	44	16.25	18.7	Short migrant
Song Thrush	15	11	10	15	51	12	8	10	14	44	67.74	30.7	Short migrant
Wood Pigeon	13	8	9	9	39	10	8	7	7	32	490	41.2	Short migrant
Firecrest	14	4	8	10	36	13	3	6	9	31	5.6	22	Short migrant
Crested Tit	14	10	5	4	33	11	9	5	4	29	11.04	19.8	Resident
Great Spotted Woodpecker	9	7	6	8	32	7	8	7	8	30	74.94	29.3	Resident
Blue Tit	3	5	5	18	31	3	5	5	11	24	11.1	21	Short migrant
Eurasian Treecreeper	10	7	6	1	24	9	5	6	1	21	9	19.2	Resident
Eurasian Jay	5	6	7	4	22	4	6	4	4	18	159.46	19.2	Short migrant
Eurasian Siskin	2	5	2	4	13	2	3	1	4	10	13.24	36.5	Short migrant
Red Crossbill	7	2	1	3	13	5	1	1	3	10	38.29	37.6	—
Greenfinch	2	1	4	5	12	2	1	4	4	11	26	32.5	Short migrant
Short‐toed Treecreeper	4	4	0	4	12	3	4	0	4	11	8.2	17.7	Resident
Eurasian Bullfinch	3	2	4	1	10	3	2	3	1	9	24.26	24.9	Short migrant
Eurasian Nuthatch	2	5	0	3	10	2	4	0	3	9	20.37	23.6	Resident
Carrion Crow	1	0	1	7	9	1	0	1	5	7	570	36.9	Short migrant
Common Redstart	1	0	3	4	8	1	0	1	3	5	14.59	25.8	Tropical migrant
Dunnock	0	1	4	2	7	0	1	4	2	7	20.24	21.2	Short migrant
Eurasian Skylark	0	0	0	7	7	0	0	0	5	5	37.31	34.7	Short migrant
Fieldfare	0	0	0	6	6	0	0	0	4	4	106	33.3	Short migrant
Goldfinch	2	0	1	2	5	1	0	1	2	4	16	33.4	Short migrant
Long‐tailed Tit	0	2	1	2	5	0	2	1	2	5	8.6	21.6	Resident
Common Buzzard	2	0	1	1	4	1	0	1	1	3	759.1	38.2	Short migrant
Hawfinch	0	0	2	2	4	0	0	2	2	4	56.63	32.9	Short migrant
Marsh Tit	1	0	1	1	3	1	0	1	1	3	11.14	17.9	Resident
Spotted Flycatcher	1	1	0	1	3	1	1	0	1	3	15.9	30.2	Tropical migrant
Willow Warbler	0	0	1	2	3	0	0	1	2	3	8.7	25.9	Tropical migrant
Yellowhammer	0	0	0	3	3	0	0	0	3	3	29.7	25.7	Short migrant
Black Redstart	0	0	1	1	2	0	0	1	1	2	16.5	23.1	Short migrant
Black Woodpecker	1	0	1	0	2	1	0	1	0	2	321	26.4	Resident
Common Raven	1	0	1	0	2	1	0	1	0	2	927.97	39.8	Resident
Garden Warbler	0	1	0	1	2	0	1	0	1	2	18.2	30	Tropical migrant
Green Woodpecker	0	1	0	1	2	0	1	0	1	2	176	25.3	Resident
Eurasian Magpie	0	0	1	0	1	0	0	1	0	1	217.48	22.9	Resident
Carrion Crow	0	0	0	1	1	0	0	0	1	1	570	36.9	Short migrant
Rook	0	0	0	1	1	0	0	0	1	1	452.1	41	Short migrant
Willow Tit	0	0	1	0	1	0	0	1	0	1	11.1	17.7	Resident

^a^ (Tobias et al. 2022).

## Appendix 3

NMDS representation of the visits colored by type of plot with the species representation in the NMDS, Considering the Jaccard Index of Dissimilarity. The ellipses represent the 95% confidence interval of each plot type's centroids.
