# Supplementary material for: Effects of Linear Openings in Forests on Temperate Bird Communities
Source: Ecol Evol. 2025 Nov 16;15(11):e72466. doi: 10.1002/ece3.72466 (PMC12620058; doi:10.1002/ece3.72466)
Supplement: Supplementary file 1 — Figure S1: Bird abundances per plot types analysis. a. Tukey's HSD post hoc tests on the total abundance per type of plot, with the plot as a random effect. The model uses a Poisson regression. Boxplots are the observed as total abundances. Error bars represent (estimated marginal) means with 95% confidence interval for each type of plot the Tukey's HSD post hoc test shows no effect of the type of plots on the total abundance. Letters “a” and “b” next to the bars indicate significant differences between groups, where groups sharing the same letter are not significantly different from each other. Note that the estimated marginal means (dots) can be different from the medians (bar in the boxplots) in the observed indices. b. Pairwise comparisons in the Tukey's HSD post hoc test on the total abundance depending on the type of plots. Figure S2: Pairwise comparisons in Tukey's HSD post hoc depending on the plot type on the a. Species richness and b. Shannon Index (associated with Figure 2a,b, respectively). Figure S3: Tukey's HSD post hoc test on the effects of the pair of types of plots on the dissimilarity with the Jaccard Index of Dissimilarity on presence/absence data, with the pair of plots as a random effect, using normal distributions (ANOVA with mixed effects). Boxplots are the observed Jaccard dissimilarities. Red dots and error bars represent (estimated marginal) means with 95% confidence interval per group. Letters “a” and “b” next to the bars indicate significant differences between groups, where groups sharing the same letter are not significantly different from each other. Figure S4: Ranks in the ANOSIM between points among groups compared to the overall ranks plot. Figure S5: Pairwise comparisons in the Tukey's HSD post hoc test on the weighted mean on the a. Body Mass and b. HWI for each pair of plot types. [file ECE3-15-e72466-s001.docx]

# Supporting information


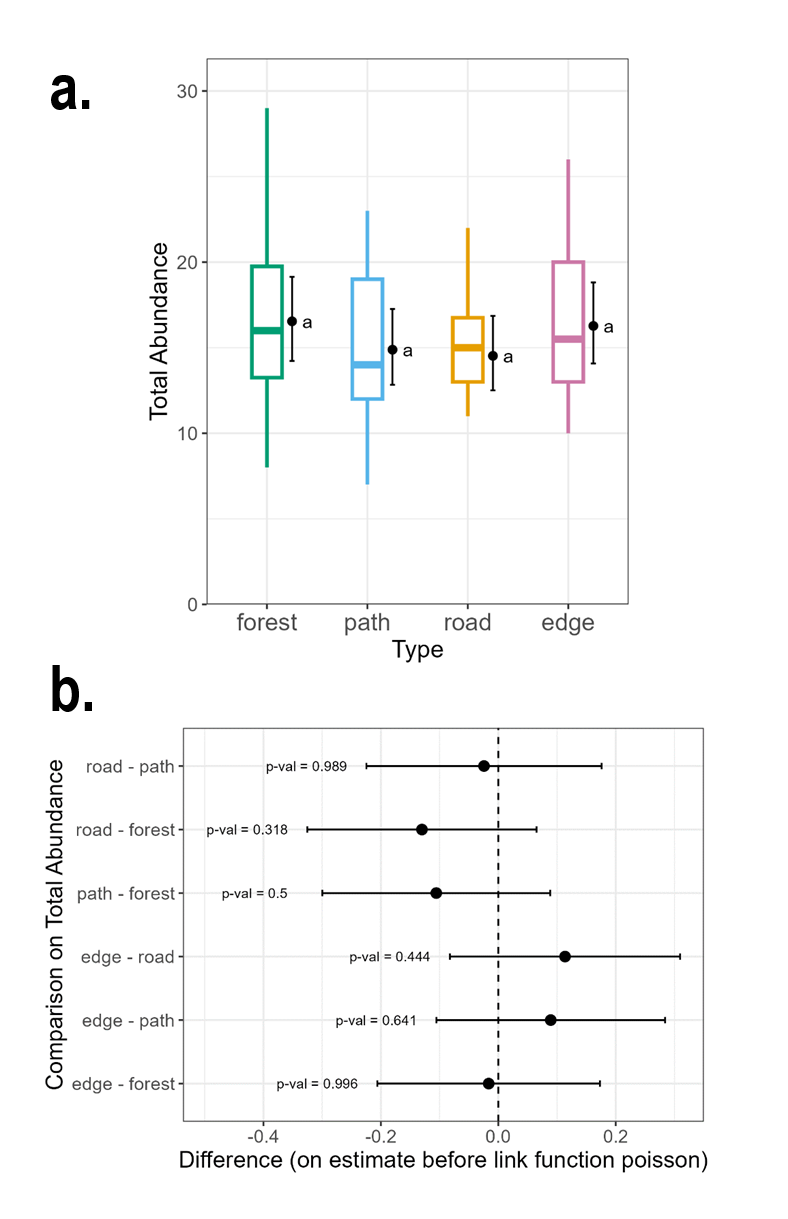


**Figure S1:** Bird abundances per plot type analysis. a. Tukey’s HSD post hoc tests on the total abundance per type of plot, with the plot as a random effect. The model uses a Poisson regression. Boxplots are the observed as total abundances. Error bars represent (estimated marginal) means with 95% confidence interval for each type of plot. The Tukey’s HSD post-hoc test shows no effect of the type of plots on the total abundance. Letters “a” and “b” next to the bars indicate significant differences between groups, where groups sharing the same letter are not significantly different from each other. Note that the estimated marginal means (dots) can be different from the medians (bar in the boxplots) in the observed indices. b. Pairwise comparisons in the Tukey’s HSD post hoc test on the total abundance depending on the type of plots.


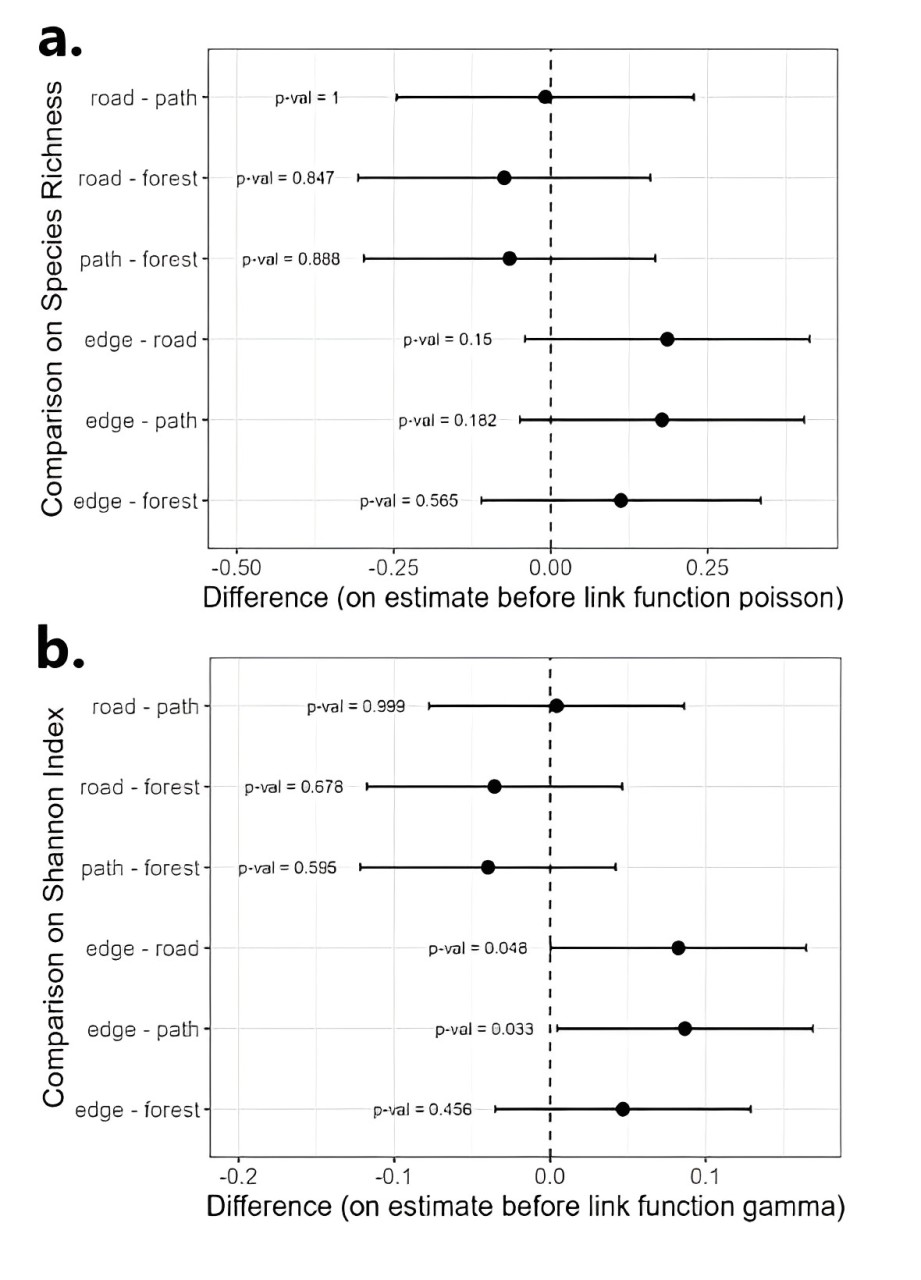


**Figure S2:** Pairwise comparisons in Tukey’s HSD post hoc depending on the plot type on the a. Species richness and b. Shannon Index (associated with Figure 2a,b, respectively).


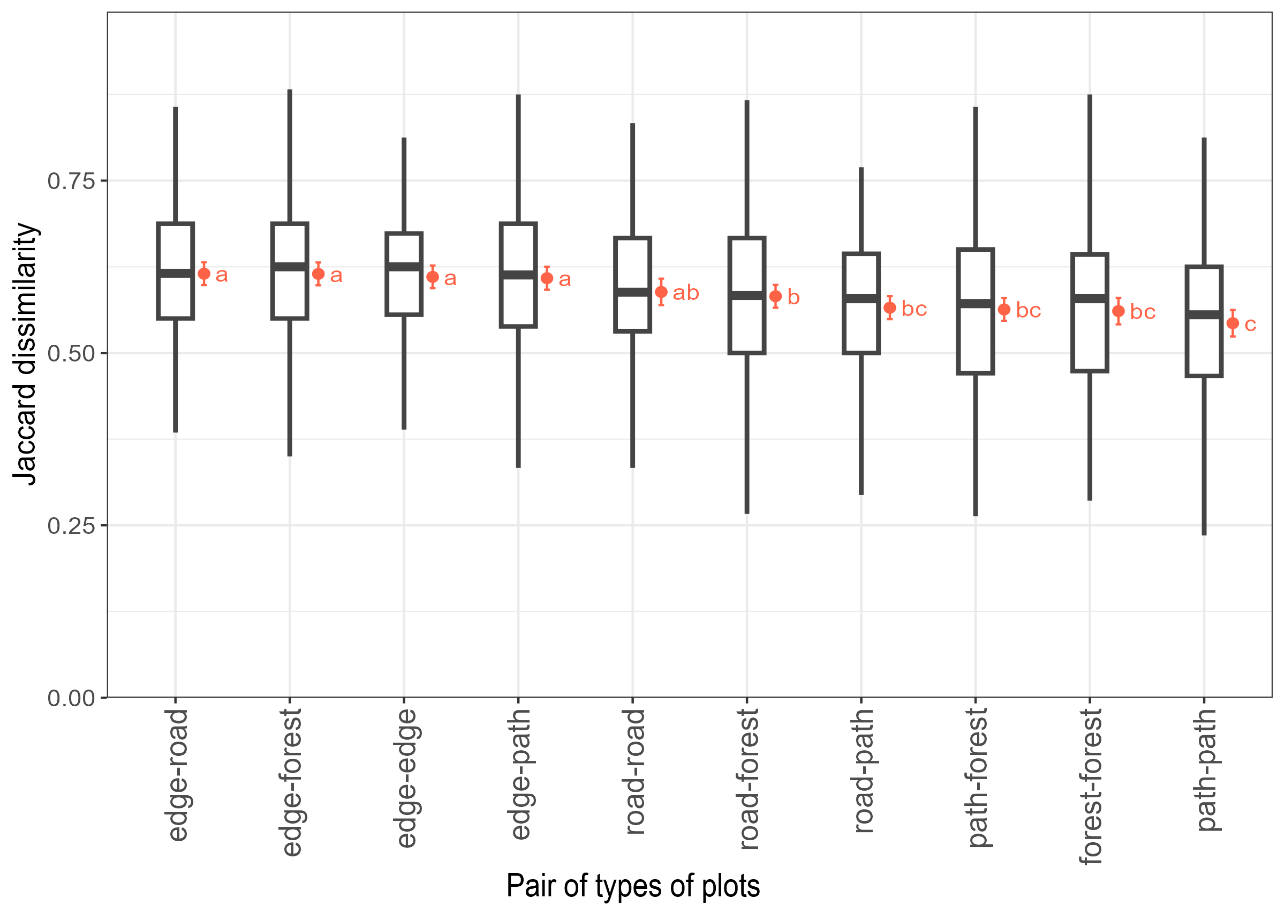


**Figure S3:** Tukey’s HSD post hoc test on the effects of the pair of types of plots on the dissimilarity with the Jaccard Index of Dissimilarity on presence/absence data, with the pair of plots as a random effect, using normal distributions (ANOVA with mixed effects). Boxplots are the observed Jaccard dissimilarities. Red dots and error bars represent (estimated marginal) means with 95% confidence interval per group. Letters “a” and “b” next to the bars indicate significant differences between groups, where groups sharing the same letter are not significantly different from each other.


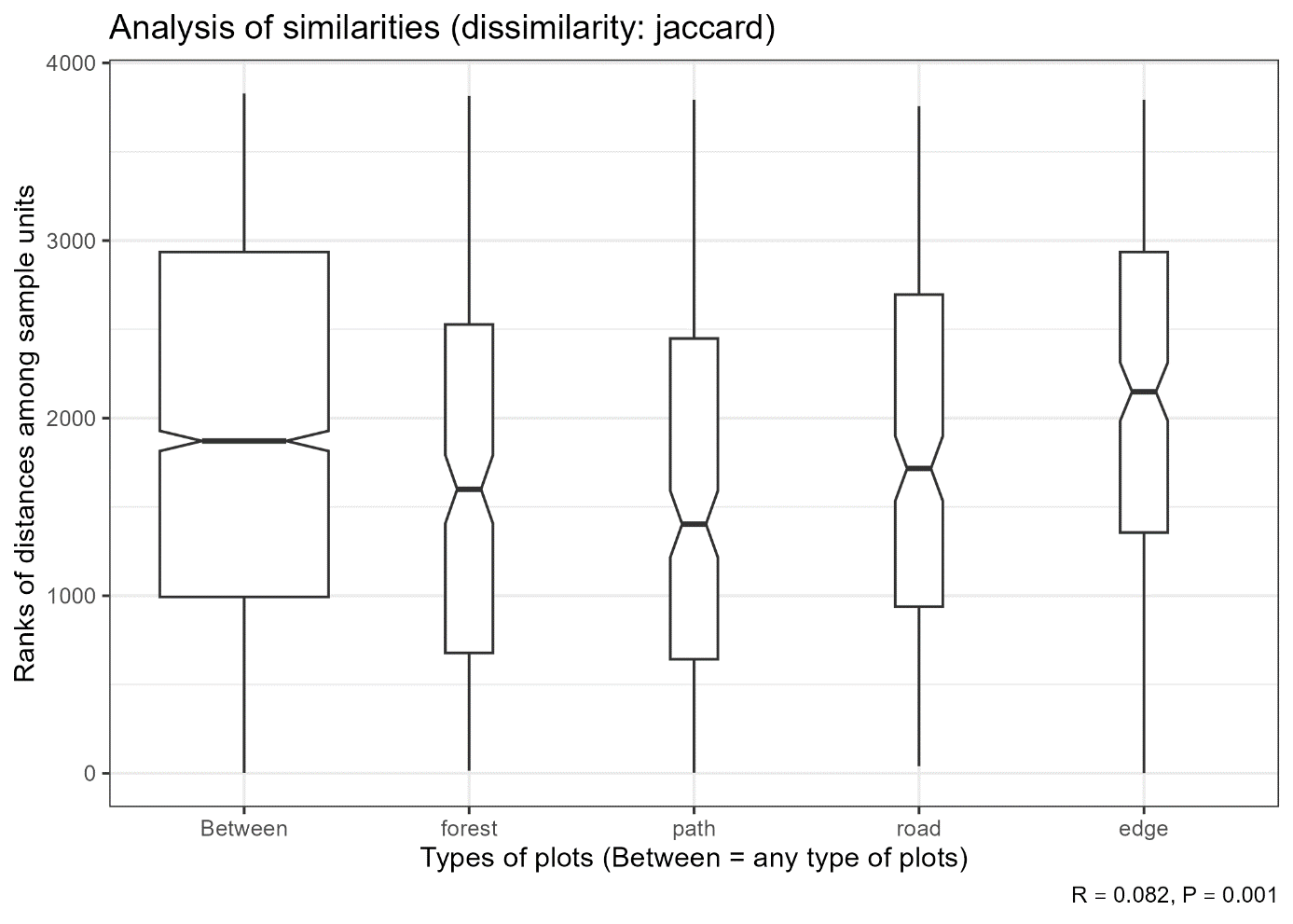


**Figure S4:** Ranks in the ANOSIM between points among groups compared to the overall ranks plot.


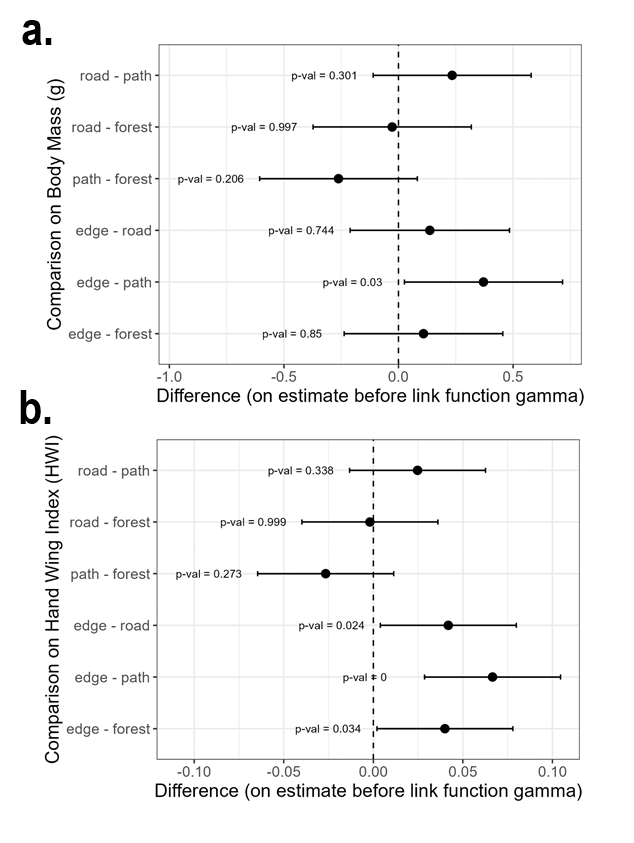


**Figure S5:** Pairwise comparisons in the Tukey’s HSD post hoc test on the weighted mean of a. Body Mass and b. HWI for each pair of plot types.
